# The cedar project: using indigenous-specific determinants of health to predict substance use among young pregnant-involved aboriginal women

**DOI:** 10.1186/s12905-017-0437-4

**Published:** 2017-09-15

**Authors:** Sana Z. Shahram, Joan L. Bottorff, Nelly D. Oelke, Leanne Dahlgren, Victoria Thomas, Patricia M. Spittal, For the Cedar Project Partnership

**Affiliations:** 1 Faculty of Health and Social Development, University of British Columbia, 1147 Research Road, Kelowna, BC V1V 1V7 Canada; 2 Institute for Healthy Living and Chronic Disease Prevention, and School of Nursing, Faculty of Health and Social Development, University of British Columbia, 1147 Research Road, Kelowna, BC V1V 1V7 Canada; 3 Faculty of Health Sciences, Australian Catholic University, Melbourne, Australia; 4 School of Nursing, Faculty of Health and Social Development, University of British Columbia, 1147 Research Road, Kelowna, BC V1V 1V7 Canada; 5 Department of Obstetrics & Gynaecology, Faculty of Medicine, University of British Columbia, 1190 Hornby Street 4th Floor, Vancouver, BC V6Z 2K5 Canada; 6 Wuikinuxv Nation, The Cedar Project, Vancouver, Canada; 7 School of Population and Public Health, University of British Columbia, 2206 East Mall, Vancouver, BC V6T 1Z3 Canada; 8 Present Address: Postdoctoral Research Fellow, Centre for Addictions Research of British Columbia, University of Victoria, PO Box 1700, STN CSC Victoria, Victoria, BC V8W 2Y2 Canada; 9 15890 Greenhow Road, Oyama, BC V4V 2E6 Canada

**Keywords:** Canada, Aboriginal health, Women’s Health, Substance use, Addictions, Pregnancy, Maternal health, Social determinants of health

## Abstract

**Background:**

Indigenous women in Canada have been hyper-visible in research, policy and intervention related to substance use during pregnancy; however, little is known about how the social determinants of health and substance use prior to, during, and after pregnancy intersect. The objectives of this study were to describe the social contexts of pregnant-involved young Indigenous women who use substances and to explore if an Indigenous-Specific Determinants of Health Model can predict substance use among this population.

**Methods:**

Using descriptive statistics and hierarchical logistic regression guided by mediation analysis, the social contexts of pregnant-involved young Indigenous women who use illicit drugs’ lives were explored and the *Integrated Life Course and Social Determinants Model of Aboriginal Health*’s ability to predict heavy versus light substance use in this group was tested (*N* = 291).

**Results:**

Important distal determinants of substance use were identified including residential school histories, as well as protective factors, such as sex abuse reporting and empirical evidence for including Indigenous-specific determinants of health as important considerations in understanding young Indigenous women’s experiences with pregnancy and substance use was provided.

**Conclusions:**

This analysis provided important insight into the social contexts of women who have experiences with pregnancy as well as drug and/or alcohol use and highlighted the need to include Indigenous-specific determinants of health when examining young Indigenous women’s social, political and historical contexts in relation to their experiences with pregnancy and substance use.

## Background

In Canada, Aboriginal^1^ ^2^ mothers have been hyper-visible in research, policy and intervention related to alcohol and drug use during pregnancy, while the social contexts underlying Aboriginal women’s substance use have often been ignored [17, 22] particularly as they relate to experiences with pregnancy. Due to imposed legislative and social conditions, beginning with colonization, many young Aboriginal mothers are located at the intersections of multiple dimensions of social inequality that shape their experiences with substance use and parenting in complex ways. However, there is a dearth of epidemiological data that explores these contextual factors related to substance use before, during and after pregnancy, and quantitative data which necessarily and explicitly attends to understanding these broader determinants of substance use is needed [22]. To understand these determinants across women’s lives in a more nuanced and contextualized way, the typical research focus on substance use only *during* pregnancy must be broadened to include pregnant-involved women’s life experiences with alcohol and drug use before, during and after pregnancy. For the purposes of this research project, pregnant-involved was defined as having ever experienced a pregnancy, regardless of pregnancy outcome or subsequent mothering role.

### The social determinants of substance use

Heavy and frequent substance use by women typically peaks among women aged 18 to 24 years old, and is highest among women with lower incomes and/or lower levels of education [1]. Women who use alcohol or drugs problematically are also often living in high risk environments characterized by poverty, unstable housing, food insecurity and unemployment, and often have histories of abuse and psychological issues [17].

Aboriginal women who use substances often face triple discrimination and marginalization as women, Aboriginal people, and people who use substances [7]. When compared to the rest of Canada, the comparatively young population of Aboriginal peoples bear a disproportionate burden of illness, poor health and violent life experiences [6, 14], while also experiencing higher unemployment rates, and lower formal education attainment and incomes (with Aboriginal women having lower incomes than Aboriginal men) [5, 4, 18]. While Aboriginal young women have higher rates of problematic substance use in Canada than non-Aboriginal young women [17], the contexts of use are explicitly linked to these contemporary health and social inequities that are the downstream manifestations of the colonial process (including social and cultural disruption, and historical and intergenerational trauma) that continues to impact Aboriginal peoples lives today [14, 15].

### The integrated life course and social determinants of aboriginal health (ILCSD) model

In 2009, the National Collaborating Centre for Aboriginal Health commissioned a report on the health inequalities experienced by Indigenous peoples in Canada, which also introduced *The Integrated Life Course and Social Determinants Model of Aboriginal Health (ILCSD) “*as a promising conceptual framework for understanding the relationships between social determinants and various health dimensions, as well as examining potential trajectories of health across the life course” (p.6, [19]). Importantly, the ILCSD locates Indigenous health outcomes within the socio-political context of being Indigenous in Canada and in relation to the nested influences of distal (i.e. social, political and historical contexts), intermediate (i.e. health, education and community infrastructure and systems, environmental stewardship and cultural continuity) and proximal (i.e. physical, mental, emotional or spiritual health impacts) determinants, across the life course. The *ILCSD model* provides an opportunity to explore social determinants not previously examined in the epidemiological literature focusing on pregnant-involved Aboriginal women [22].

A better understanding of the social determinants underlying pregnant-involved Aboriginal women’s substance use is needed to inform policies and programs. The research questions guiding this study were:

This research study aimed to answer the following questions:

1. What are the social contexts of the lives of pregnant-involved young Aboriginal women who use alcohol and drugs in British Columbia, Canada?
2. Can the *ILCSD Model’s* social determinants of health within distal, intermediate and proximal domains predict heavy alcohol use, drug use (smoked) and drug use (injected) in the previous six months among pregnant-involved young Aboriginal women?

Hypothesis #1: The influence of distal determinants on each dependent variable (alcohol use, drug use (smoked) and drug use (injected), will be mediated by intermediate and proximal determinants.

Hypothesis #2: The influence of intermediate determinants on each dependent variable (alcohol use, drug use (smoked) and drug use (injected), will be mediated by proximal determinants.

## Methods

A secondary data analysis was conducted using data from a baseline questionnaire that was administered in a larger project, the Cedar Project, to all participants at enrollment [25]. A descriptive quantitative design was used, in addition to hierarchical logistic regression guided by mediation analysis principles, to test the *ILCSD Model’s* ability to predict heavy substance use among pregnant-involved female participants.

### Data and study setting

A secondary data analysis using survey data from a larger research study, The Cedar Project, was conducted. The Cedar Project is an ongoing prospective cohort study of young Aboriginal men and women who use drugs in three centres in British Columbia, Canada [25] (Table 2). The Cedar Project’s purpose is to explore HIV- and HCV- related vulnerabilities among male and female Aboriginal youth who use drugs. Recruitment for the project began in October 2003 and is ongoing. Participants are recruited through health care providers, street outreach workers, and word of mouth. Eligibility criteria for the Cedar project included self-identification as Aboriginal, being between the ages of 14–30 years of age, and having smoked illicit drugs in the last week, or injected illicit drugs in the last month, including crystal methamphetamine, crack-cocaine, heroin or cocaine, prior to enrolment. Saliva screens were used to confirm drug use. Table 1 shows a comparison of the three study sites for several relevant factors related to the lives of pregnant-involved young Aboriginal women who live there.

**Table 1:** Comparison of study site characteristics

Characteristic	Vancouver (Site A)	Prince George (Site B)	Interior (Site C)
Urban/Rural Mix	Large Urban Centre	Small Urban Centre	Urban-Rural Mix
Harm Reduction vs. Abstinence Service Models	Primarily Harm Reduction	Harm Reduction	Primarily Abstinence-Based
Aboriginal Population*	40,310 (2% of total)	8855 (11% of total)	7050 (7.7% of total)
On or Off- Reserve Living	Primarily off-reserve	Primarily off-reserve	Mixture
Service Density	Dense in downtown eastside	Dense in downtown core	Dense in Kamloops, Sparse everywhere else

Based on 2006 Statistics Canada Census Data for Greater Vancouver, Prince George, and Kamloops

Data collection procedures for the Cedar Project have been detailed elsewhere [25]. This analysis is based on the baseline questionnaire that is administered at enrollment to all Cedar Project participants to elicit socio-demographic characteristics, patterns of drug use, sexual vulnerability, use of services and to assess the risk factors associated with Aboriginal youth’s elevated risk and transmission of HIV and HCV.

### Cohort definition

In order to understand women’s life contexts and experiences with alcohol and drug use before, during and after pregnancy, this secondary analysis was restricted to “pregnant-involved women” defined as women who have ever been pregnant before the age of 30. Not restricting the sample to women who were currently pregnant, or any defined outcome of pregnancy, was a purposeful decision to explore women’s life experiences with substances and pregnancy more fully, while rejecting the notion that women’s health is only of import if it relates to the health of a foetus or child.

For this analysis, the cohort was defined as all female participants under the age of 30 years who completed a baseline questionnaire between October 2003–July 2013 and responded ‘yes’ to the question ‘Have you ever been pregnant?’ The resulting study sample was 291. Anonymized data that included the following measures was available for analysis.

### Measures

Based on the *ILCSD Model*, indicators were selected that were deemed most relevant in measuring the proximal, intermediate or distal social determinants of health. Variables available for this analysis included measures of socio-demographic factors, pregnancy characteristics, survival sex^3^ involvement, sexual abuse histories, cultural continuity, the use of health care services, alcohol and/or drug treatment services, the use of any services in general, and measures of colonialism and historical or cultural trauma. Table 2 shows a summary of all included variables, as well as their definitions for further clarification.

**Table 2:** Self-Report Variable Classifications according to ILCSD Model and Definitions

Variable names	Variable definitions
Proximal Determinants	
Socioeconomic Status (SES)	
Relationship Status	Current relationship status.
Highest Education	Highest level of education completed.
Income	Monthly income from all sources (gov’t, work, and illegal sources).
Survival Sex, ever	Has the participant ever done survival sex work?
IF YES,	
Age of 1st Survival Sex	Age of participant the first time she did survival sex work.
Survival Sex, last 6 Months	Has the participant done survival sex work in the previous 6 months?
Physical Environments	
Housing Stability	Considered unstable if lived anywhere other than house or apartment in previous 6 months (i.e. hotel, hostel, shelter, crack shack etc.).
Homelessness	Has the participant ever been on the street with no place to sleep for more than three nights?
Age First Left Home	Age the participant first left home to live on her own.
Health Behaviours	
Number of Pregnancies	Number of times the participant has ever been pregnant (including abortions/miscarriages).
Age of First Pregnancy	Age of participant the first time she was pregnant.
Trauma	
Sexual Abuse, ever	Has the participant ever been sexually abused? (Any type of forced sexual activity including childhood sexual abuse, molestation, rape, and sexual assault)
IF YES,	
Age of 1st Sexual Abuse	Age of participant the first time she was sexually abused.
Sexual Abuse, reported	Has the participant ever reported the sexual abuse to anyone?
Sexual Abuse, repeated	Has the participant been sexually abused again, since the first time?
Sexual Abuse, last 6 Months	Has the participant been sexually abused in the previous 6 months?
Mothering Experiences	
Child Apprehended, ever	Has the participant ever had any of her children apprehended by child and family services?
Intermediate Determinants	
Cultural Continuity	
Taken from Parents, ever	Has the participant ever been taken from her biological parents by child and family services?
IF YES,	
Age 1st Taken from Parents	Age of participant the first time she was taken from her biological parents.
Language	Does the participant speak her native or traditional language?
Reserve, ever	Has the participant ever been to a reserve?
Cultural Substance Treatment	Is the participant interested in more culturally specific substance use treatment?
Services Used within the previous 6 months	
Emergency Room Visit	Has the participant received health care from the emergency room (ER) in the previous 6 months?
Admitted to Hospital	Has the participant been admitted overnight to a hospital in the previous 6 months?
Ambulance	Has the participant received health care from an ambulance in the previous 6 months?
	Has the participant ever received any substance abuse treatment (including methadone)?
Counselling Services	Has the participant accessed a counsellor in the previous 6 months?
Food Services	Has the participant accessed food services in the previous 6 months?
Visit with a Health Care Provider Visit	Has the participant accessed a health care provider in the previous 6 months?
Housing Services	Has the participant accessed housing services in the previous 6 months?
Needle Exchange Services	Has the participant accessed a needle exchange in the previous 6 months?
Support Group Services	Has the participant accessed a support group in the previous 6 months?
Social Worker	Has the participant accessed a social or welfare worker in the previous 6 months?
Service Barriers	
Housing Denied, due to drug Use	Has the participant ever had housing denied due to her drug use?
Service Denied, due to drug use	Has the participant ever had a service denied due to her drug use?
Barriers to Services	Does the participant feel there are barriers to accessing services she needs?
Service Needs	
Service Needed, last 6 months	Has the participant been in need of any service, in the previous 6 months?
Distal Determinants	
Colonialism	
Residential School, parents	Has either of the participant’s parents attended residential school?
Residential School, family History	Has anyone in the participant’s family (excluding parents) attended residential school?
Number of Family Members	Number of known family members (excluding parents) who attended residential school
Caregiver Addiction	Did any of the participant’s caregivers have drug or alcohol addiction problems?
Survival Sex History	Did anyone in the participant’s family do survival sex work?

#### Dependent variables

Three dependent variables were used that measured the participants’ pattern of alcohol use, drug use (smoked) and drug use (injected) over the previous 6 months, respectively. Based on previous studies of people who use illicit drugs [9], heavy drug smoking or injecting was defined as those who reported smoking or injecting once or more per day and light drug use was defined as using less than daily (heavy vs. light use). Alcohol use over the previous 6 months was defined as heavy for participants who reported having 6 or more drinks on one occasion on more than a monthly basis, and light for participants having 6 or more drinks on one occasion once a month or less, based on the low risk drinking guidelines from the Canadian Centre on Substance Abuse and the information available in the survey about alcohol use patterns [8] (heavy vs. light use).

**Fig. 1:**
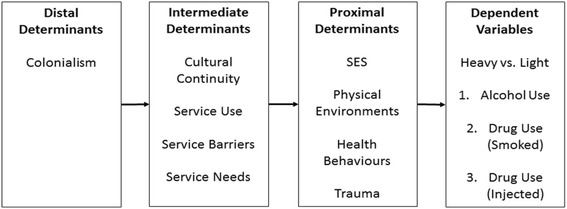
Hypothesized relationship between variables based on the Integrated Life Course and Social Determinants Model of Aboriginal Health

Given that all the participants were women who used drugs at enrollment, creating outcome variables to distinguish between light and heavy use allowed for an exploration of the relationships between social determinants of health and substance use. This was also particularly relevant given that pregnant-involved women who have a history of heavy drug and/or alcohol use are more likely to use alcohol and/or drugs during pregnancy, and also, heavy use of substances during pregnancy specifically, is associated with greater harms for both the mother and the foetus [15, 21]. While this variable measures level of use within the past 6 months and, therefore, is not measuring use during a pregnancy necessarily, it is nonetheless an important and relevant measure to examine the impact of the social determinants of health on substance use among pregnant-involved young Aboriginal women. Figure 1 depicts the hypothesized relationship between the distal, intermediate and proximal determinants of health, and the three dependent variables.

### Data analysis

Descriptive statistics were used to describe the sample. Categorical variables were compared across the three study locations of the project using Pearson’s *x* ^2^ test. No expected cell values were less than 5. Continuous variables were analyzed using the Kruskal-Wallis one-way analysis of variance for non-parametric data. All reported *p*-values are two-sided and significant associations were determined at the 0.05 cut-off point. Continuous variables were inspected for outliers, and outliers were replaced with the value of two times the variable’s standard deviation. Multicollinearity and linearity of the logit was also inspected before conducting logistic regressions.

Univariate logistic regression was conducted to identify the determinants of health that were independently associated with each of the outcome measures. In the adjusted Model I for each dependent variable, significant variables at the *p* < 0.05 cut-off in univariate analysis were entered into multivariable logistic regression analysis using the Enter method in SPSS. All models were adjusted for age.

In the adjusted Model II for each dependent variable, variables that remained significant in Model I were entered as blocks according to their hypothesized relationship based on the *ILCSD Model* to test for any mediated effects to support the model*.* The most common method for testing mediation involves four steps: First, there is shown to be a significant relationship between a predictor and outcome; second, there is shown to be a significant relationship between the mediator and the predictor; third, there is shown to be a significant relationship between the mediator and the outcome; and, fourth, there is shown to be a significant reduction in the strength of the relationship between the predictor and the outcome when the mediator is added to the model [3, 13]. In this analysis, there were no significant relationships between any predictor variables and hypothesized mediator variables (step two of mediation analysis), so further mediation analysis was not possible beyond showing the results of the full models in Model II. Both unadjusted and adjusted odds ratios and 95% confidence intervals were obtained using logistic regression.

## Results

The sample for this secondary analysis included 291 pregnant-involved young Aboriginal women: 154 (52.9%) completed their baseline questionnaires in Vancouver, 111 (38.1%) completed their baseline questionnaires in Prince George, and 26 (9%) completed their baseline questionnaires in the Interior region of British Columbia. The median age of participants was 24 years old, and the majority of participants were single (64.4%), had not completed high school (79.5%), and were living in unstable housing situations (66.2%). Also, the majority of women had ever been homeless (65.9%), while 67.7% of women had ever been sexually abused and the median age of first sexual abuse was six years old (range 1–20 years old).

### Descriptive results

Table 3 shows comparisons of all the included social determinants of health based on location of the participant, and addresses the first research question of this study. Participants in Vancouver were older and more likely to have ever been homeless, while participants in both Vancouver and Prince George were more likely than Interior participants to have been taken from their biological parents, to have participated in survival sex ever or in the last six months, to have lower monthly incomes, to be interested in culturally specific treatment options, and to have accessed a needle exchange or a social/welfare worker in the last six months. Vancouver and Prince George participants were less likely than Interior participants to speak a traditional language, to have visited the emergency room or have been treated by an ambulance in the past six months, and to have accessed a counsellor in the last six months. Participants in Prince George left home for the first time at a younger age, and were more likely to state that they had needed any social or health service or had accessed housing services in the previous 6 months.

**Table 3:** Comparison of Proximal, Intermediate and Distal Determinants between Participants in Vancouver, Prince George and the Interior

Characteristic	Vancouver (*n* = 154) n (%)	Prince George (*n* = 111) n (%)	Interior (*n* = 26) n (%)	*p-*value	Total (%) (*N* = 291) n (%)
Proximal Determinants					
Median age at enrollment, years (range)	24 (16–30)	23 (15–30)	23 (16–30)	0.024	24 (15–30)
Single	107 (69.9)	67 (60.4)	12 (48)	0.280	186 (64.4)
Did not complete high-school	121 (79.1)	84 (77.1)	24 (92.3)	0.220	229 (79.5)
Median monthly income, dollars (range)	558 (80–13,000)	850 (40–10,100)	1035 (100–5000)	0.023	748 (40, 30,000)
Survival sex, ever	116 (76.8)	77 (72)	10 (38.5)	<0.001	203 (71.5)
IF YES, (*n* = 203)					
Median age of first survival sex, years (range)	16 (11–28)	16 (9–27)	17 (12–23)	0.303	16 (9–28)
Survival sex, last 6 months	89 (57.8)	65 (58.6)	5 (19.2)	0.001	159 (54.6)
Unstable Housing (last 6 months)	109 (71.2)	70 (63.1)	13 (50)	0.071	192 (66.2)
Ever lived on the streets (>3 nights)	116 (75.3)	62 (56.4)	13 (50)	0.001	191 (65.9)
Median age first left home, years (range)	16 (8–22)	14 (8–19)	16 (12–21)	0.001	15 (8–22)
Median number of pregnancies (range)	2 (1–5)	2 (1–5)	2 (1–5)	0.238	2 (1–5)
Median age of first pregnancy, years (range)	17 (12–25)	17 (10–24)	18.16 (13–24)	0.068	17 (10–25)
Ever sexually abused	102 (66.2)	80 (72.1)	15 (57.7)	0.315	197 (67.7)
IF YES, (*n* = 197)					
Median age first sexually abused (years) (range)	6 (1–18)	8 (2–19)	9 (3–20)	0.057	6 (1–20)
Child apprehended, ever	72 (49.7)	47 (44.3)	8 (36.4)	0.430	127 (46.5)
Intermediate Determinants					
Ever taken from biological parent	104 (67.5)	75 (67.6)	11 (42.3)	0.036	190 (65.3)
IF YES, (*n* = 190)					
Median age first taken from biological parents (range)	4 (1–17)	5 (0–14)	6 (1–13)	0.666	5 (0–17)
Speak traditional language	21 (13.6)	22 (20)	12 (46.2)	<0.001	55 (19)
Ever been to a reserve	121 (81.2)	98 (89.1)	25 (96.2)	0.056	244 (85.6)
Interested in more culturally specific treatment	77 (50)	72 (64.9)	8 (32)	0.004	157 (54.1)
Substance Use Treatment, Ever	110 (71.4)	92 (82.9)	22 (84.6)	0.057	224 (77)
Services used within the previous 6 months					
Emergency Room Visit	49 (31.8)	53 (47.7)	15 (57.7)	0.005	117 (40.2)
Admitted to Hospital	32 (21.1)	22 (19.8)	9 (34.6)	0.245	63 (21.8)
Ambulance	37 (24)	20 (18)	12 (46.2)	0.010	69 (23)
Counselling Services	25 (16.2)	39 (35.1)	13 (50)	<0.001	214 (73.5)
Food Services	85 (55.2)	58 (52.3)	12 (46.2)	0.669	136 (46.7)
Visit with a Health Care Provider Visit	77 (50)	58 (52.3)	13 (50)	0.933	148 (50.9)
Housing Services	35 (22.7)	48 (43.2)	7 (26.9)	0.002	201 (69.1)
Needle Exchange Services	73 (47.4)	85 (76.6)	4 (15.4)	<0.001	129 (44.3)
Support Group Services	9 (5.8)	11 (9.9)	4 (15.4)	0.189	24 (8.2)
Social Worker	68 (44.2)	69 (62.2)	6 (23.1)	<0.001	148 (50.9)
Denied housing due to drug use	45 (29.4)	24 (21.6)	7 (26.9)	0.363	76 (26.2)
Denied service due to drug use	37 (24)	18 (16.2)	6 (23.1)	0.294	61 (21)
Have barriers to accessing services	13 (8.5)	7 (6.3)	4 (15.4)	0.315	24 (8.3)
Needed a service, last 6 months	100 (65.8)	93 (83.8)	18 (69.2)	0.005	211 (73)
Distal Determinants					
At least one parent attended Residential School	73 (47.4)	49 (45)	9 (34.6)	0.478	131 (45.3)
Residential School Family History	104 (68)	81 (73)	22 (84.6)	0.198	207 (71.1)
Median number of family members in Residential School (range)	4 (1–19)	3.5 (0–36)	5 (0–29)	0.648	3 (0–36)
At least one caregiver with drug or alcohol addiction	124 (81)	87 (78.4)	30 (76.9)	0.813	231 (79.7)
Family history of survival sex	50 (43.1)	39 (52)	5 (50)	0.474	94 (46.8)

For continuous variables, range is reported instead of percentage

### Testing the ILCSD model for predicting heavy versus light substance use

In order to address the second research question, the ILCSD Model’s ability to predict heavy versus light substance use was assessed for each dependent variable. If variables remained significant even after block entry according to the ILCSD model, then there was no evidence of mediation occurring.

#### Heavy versus light alcohol use

Table 4 shows the results from the univariate and multivariate logistic regression analyses conducted with pattern of alcohol use as the dependent variable. In univariate analyses, participants who lived in Vancouver and Prince George were significantly less likely to have more than six drinks in one occasion more than once a month than participants who lived in the Interior (OR 0.33, 95% CI 0.13, 0.82; OR 0.35, 95% CI 0.14, 0.88 respectively). Participants who had reported their sexual abuse to somebody, were also less likely to have more than six drinks in one occasion more than once a month (OR 0.40, 95% CI 0.22, 0.73). In Model 1, multivariate logistic regression was conducted, where both interview location and sexual abuse reporting were entered as covariates in the model. Vancouver participants were significantly less likely than participants in the Interior to use alcohol more than monthly (OR 0.30, 95% CI 0.12, 0.77) and having reported sexual abuse was also protective (OR 0.38, 95% CI 0.21, 0.71). Model II tested the *ILCSD Model* using the block entry shown in Fig. 1. Since both determinants remained statistically significant, their direct effects seem to override any mediation expected according to the ILCSD model.

**Table 4:** Univariate and Multivariate Modeling for Alcohol Use among Participants (*N* = 210)

	Monthly or less (*n* = 144) *N (%)*	More than monthly (*n* = 66) N (%)	Unadjusted OR (95% CI)	Adjusted OR Model I (95% CI)	Adjusted OR Model II (95% CI)
Location					
Interior	11 (7.6)	13 (19.9)	Reference	Reference	Reference
Prince George	61 (42.4)	25 (37.8)	0.35* (0.14, 0.88)	0.38 (0.14, 1.01)	0.38 (0.14, 1.01)
Vancouver	72 (50)	28 (42.4)	0.33* (0.13, 0.82)	0.30* (0.11, 0.78)	0.30* (0.11, 0.78)
Sex Abuse Reported					
No	55 (38.2)	40 (60.6)	Reference	Reference	Reference
Yes	89 (61.8)	26 (39.3)	0.40** (0.22, 0.73)	0.40** (0.21, 0.74)	0.40** (0.21, 0.74)
Overall Percentage Correct for Adjusted Models				70.5	70.2

*p < .05, **p < .01

#### Heavy versus light smoked drug use

Table 5 shows the results from the univariate and multivariate logistic regression analyses conducted with pattern of smoked drug use as the dependent variable. In univariate analyses, daily or more use of smoked drugs was independently associated with living in Vancouver, being single, having unstable housing, having more pregnancies, having your first pregnancy at a younger age, having participated in survival sex ever or in the last six months, having been denied a service due to drug use in the last six months, and have had either parent attend residential school. In Model I, all variables that were statistically significant at the 0.05 cut-off were entered into the logistic regression as covariates. In this model daily or more use of smoked drugs was independently associated with being single (OR 2.36, 95% CI 1.09, 5.08), having unstable housing (OR 2.17, 95% CI 1.03, 4.58), and having had either parent attend residential school (OR 4.10, 95% CI 1.17, 14). In Model II, statistically significant variables from Model I were entered in blocks as shown in Fig. 1 to test the *ILCSD Model*. All variables remained significantly associated, suggesting no evidence of mediation.

**Table 5:** Univariate & Multivariate Modeling for Drug Use (Smoked) among Participants (*N* = 285)

	< Daily (*n* = 49) *N (%)*	≥ Daily (*n* = 236) N (%)	Unadjusted OR (95% CI)	Adjusted OR Model I	Adjusted OR Model II
Location					
Interior	9 (18.4)	16 (6.8)	Reference	Reference	–
Prince George	24 (49)	86 (36.4)	2.01 (0.79, 5.13)	1.11 (0.33, 3.71)	–
Vancouver	16 (32.7)	134 (56.8)	4.71** (1.79, 12.29)	3.45 (1.00, 12.00)	–
Relationship Status					
Legally Married	24 (49)	66 (28.1)	Reference	Reference	Reference
Common Law	3 (6.1)	2 (0.9)	0.24 (0.04, 1.54)	0.15 (0.01, 2.00)	0.26 (0.03, 1.93)
Widowed/Separated/Divorced	2 (4.1)	3 (1.3)	0.55 (0.09, 3.47)	0.93 (0.06, 12.47)	0.53 (0.08, 3.58)
Single	20 (40.8)	164 (69.8)	2.98** (1.54, 5.76)	2.40* (1.11, 5.20)	3.08** (1.58, 6.02)
Housing Stability, last 6 months					
Stable	25 (51)	71 (30.2)	Reference	Reference	–
Unstable	24 (49)	164 (69.8)	2.41** (1.29, 4.5)	2.02 (0.95, 4.31)	–
Median Number of Pregnancies					
Number (SD)	2 (1.3)	2 (1.4)	1.29* (1.02, 1.63)	1.46 (1.00, 2.13)	–
Median Age of First Pregnancy					
Years (SD)	18 (2.6)	17 (2.6)	0.89* (0.77, 0.98)	0.92 (0.78, 1.09)	–
Sex Work, Ever					
No	22 (45.8)	58 (25)	Reference	Reference	–
Yes	26 (54.2)	174 (75)	2.54** (1.34, 4.82)	1.54 (0.54, 4.42)	–
Sex Work, last 6 months					
No	32 (65.3)	96 (40.7)	Reference	Reference	–
Yes	17 (34.7)	140 (59.3)	2.75** (1.44, 5.22)	1.77 (0.61, 5.13)	–
Service Denied due to Drug Use, last 6 mos					
No	45 (91.8)	181 (76.7)	Reference	Reference	–
Yes	4 (8.2)	55 (23.3)	3.42* (1.18, 9.93)	3.04 (0.97, 9.54)	–
Residential School, Parents					
No	31 (63.3)	125 (53.4)	Reference	Reference	Reference
Yes, one	4 (8.2)	53 (22.6)	3.29* (1.11, 9.77)	4.12* (1.20, 14.20)	3.67* (1.21, 11.45)
Yes, both	14 (28.6)	56 (23.9)	0.99 (0.49, 2.01)	1.00 (0.42, 2.36)	0.99 (0.47, 2.08)
Overall Percentage Correct For Adjusted Models				87.0	83.2

*p < .05, **p < .01

#### Heavy versus light injected drug use

Table 6 shows the results from the univariate and multivariate logistic regression analyses conducted with pattern of injection drug use as the dependent variable. In univariate analyses, daily or more use of injection drugs was independently associated with a higher number of pregnancies, survival sex in the last six months, and having ever received treatment. Having received sexual abuse counselling, attending support groups in the last six months, and having experienced barriers to services in the last six months were all protective. In Model I, all variables that were statistically significant at the 0.05 cut-off were entered into the logistic regression as covariates. In Model 1, having ever received substance use treatment and number of pregnancies were no longer significantly associated with daily or more injection drug use. In Model II, statistically significant variables from Model I were entered in blocks as shown in Fig. 1 to test the ILCSD Model. All variables remained significantly associated, suggesting no evidence of mediation.

**Table 6:** Univariate and Multivariate For Drug Use (Injected) among Participants (*N* = 184)

	< Daily (*n* = 76) *N (%)*	≥ Daily (*n* = 108) N (%)	Unadjusted OR (95% CI)	Adjusted OR Model 1	Adjusted OR Model 2
Median Number of Pregnancies (SD)	3 (1.46)	2 (1.37)	0.77* (0.63, 0.95)	0.80 (0.62, 1.02)	–
Survival Sex, last 6 months					
No	37 (48.7)	32 (29.6)	Reference	Reference	Reference
Yes	39 (51.3)	76 (70.4)	2.25** (1.22, 4.15)	2.75** (1.13, 4.74)	2.71** (1.40, 5.23)
Sex Abuse Counselling					
No	48 (63.2)	88 (81.5)	Reference	Reference	Reference
Yes	28 (36.8)	20 (18.5)	0.39** (0.2, 0.76)	0.42* (0.20, 0.87)	0.35** (0.17, 0.71)
Substance Use Treatment, Ever					
No	12 (15.8)	31 (28.7)	Reference	Reference	–
Yes	64 (84.2)	77 (71.3)	2.15* (1.02, 4.52)	1.73 (0.77, 3.91)	–
Support Group, last 6 months					
No	66 (86.8)	104 (96.3)	Reference	Reference	Reference
Yes	10 (13.2)	4 (3.7)	0.25* (0.08, 0.84)	0.22* (0.06, 0.79)	0.20* (0.06, 0.70)
Service Barriers, last 6 months					
No	65 (85.5)	103 (96.3)	Reference	Reference	Reference
Yes	11 (14.5)	4 (3.7)	0.23* (0.07, 0.75)	0.20* (0.06, 0.72)	0.21* (0.06, 0.73)
Overall Percentage Correct for Adjusted Models				72.5	69.4

For continuous variables, standard deviation is reported instead of percentage

*p < .05, **p < .01

## Discussion

This study reports on empirical support for the importance of integrating socio-historical contexts into models of determinants of substance use and supports a counter-narrative to the current pathologizing discourse in Canada, where “Aboriginal Status” is often cited as a determinant of health on its own [16]. Instead, by using an Aboriginal-specific model, it was possible to explore how determinants that uniquely impact Aboriginal health in Canada (including residential school histories, racism, and intergenerational trauma) have differentially impacted the health status and experiences of Aboriginal women, in an appropriately nuanced and fluid manner. This is one of the first studies to evaluate an Aboriginal-specific social determinants model to identify predictors of substance use among pregnant-involved Aboriginal women. The inclusion of variables that measured the lifelong and future impacts of colonialism and cultural continuity provide a more complete picture of the social determinants of substance use, from an Aboriginal-specific perspective. Given the lack of previous research in this area that includes and explicitly acknowledges the important contexts of substance use among young pregnant-involved Indigenous women [22], this study is a timely and important contribution to the research landscape.

In testing the *ILCSD Model,* important independent risk and protective factors for heavy substance use were identified. Among participants who had ever been sexually abused, having reported sexual abuse to anyone was found to be associated with lower alcohol use. In their study, Draucker et al. [10] also found that disclosing abuse was the main way participants were able to make sense of their experiences and to heal.

Women’s substance use is often positively correlated to their partner’s use [20]. However, being in a relationship was associated with lower smoked drug use. Having spoken anecdotally with community workers and women who use injection drugs, they suggested that for many women, their partners initiate their first use of injection drugs, as well as continue to inject for them, and so there is the possibility that single women have higher use of smoked drugs because they have not progressed to injection drug use. Further research on this topic would be beneficial. The association between parental residential school attendance and increased use of smoked drugs is an indicator of the importance for understanding intergenerational trauma and the perpetuation of harms among young Aboriginal women as well as the impacts of foster care involvement, which is understood as directly linked with residential school histories.

Having participated in survival sex in the last 6 months was associated with daily or more injection drug use. This relationship could be bi-directional because women may be participating in survival sex to support their heavier use, or they may be using drugs more heavily to cope with survival sex. Also, having a higher number of pregnancies, having received sex abuse counselling and attending a support group in the last six months, were all protective against more than daily use of injection drugs. These results again suggest that attending to sexual abuse trauma can be protective, and that peer support is also a promising strategy for some women. The results also suggest that pregnancies can be protective, possibly because women reduce drug use for the pregnancies, and/or because of increased supports during pregnancy. Finally, having experienced barriers to any services (health and supportive) was also associated with lower injection drug use. This may be because those who are heavier users are less aware of or connected to services to perceive any barriers. Similarly, if those women are not accessing services as much as women who use less, then they would not have had an opportunity to encounter any barriers to services.

Women who participate in sex work in BC have reported that histories of injection drug use further compounded risks in women’s lives and added to barriers to parenting, while limited access to appropriate non-judgmental services to support their needs as women who participated in survival sex work and who used drug mitigated access to environments or services that support them as pregnant women/parents [11]. Survival sex work may therefore be confounding or contributing to the other associations with injection drug use.

The mediation relationships between determinants according to the *ILCSD Model*, were not supported, with several caveats: first, data were not collected with the intention to test this model, and variables fit into the model retrospectively did not represent all parts of the model; second, the model was not designed specifically to explain substance use or women’s health, but for overall health and wellness of Aboriginal peoples; and, lastly, the participants in this sample were a small group of street-entrenched and street-recruited women facing extraordinary risks in their day to day lives and were not representative of the larger Aboriginal women population in Canada. Conducting this analysis comparing women who do and do not use substances may provide a more robust test of the model.

Understanding Indigenous health in Canada within the context of colonial practices both past and present [12] is needed. Poverty, homelessness, housing instability, lack of education, involvement in the child welfare system, visits to the emergency room, survival sex work, and sexual abuse were all important predictors of substance use in the study sample. Given the relationship of all of these factors with historical and contemporary colonization practices (and that over 70% of the sample had a family history of residential school attendance and addiction), the intergenerational impacts of residential schooling, addiction, survival sex and trauma must foreground any deeper understanding of substance use among young Aboriginal women. Explicit attention to these factors has been decidedly absent from the literature examining substance use among Indigenous women [19], while a lack of meaningful data that captures the distinct sociopolitical, historical and geographical contexts of Indigenous women’s lives has limited discussions on these topics [2, 12].

Multiple perspectives and models, in addition to the ILCSD Model, including for example The Indigenist Stress-Coping Model (on which The Cedar Project is based) [26], will likely be needed to capture the complexity of young Indigenous women’s experiences with both pregnancy and substance use. As evidenced by this study, these contextual factors are paramount to creating a fuller understanding of substance use and pregnancy. Further, highlighting the structural and social determinants of substance use provides actionable targets for interventions that can support women and their children. Importantly, by including the variables in this analysis related to women’s socio-political-historical contexts, we were able to present a fuller depiction of women’s actual lives, in keeping with previous qualitative findings from work with this same population [23, 24]. Indeed, a common criticism of quantitative research is its inability to produce rich and contextualized data. By developing methods for capturing and measuring Indigenous-specific determinants of health, such as intergenerational trauma, foster care and racism experiences, it will be possible to provide richer and more useful empirical data to support and develop understandings in this area of research. Racism, while playing an important role in the health and well-being of Indigenous peoples in Canada [2], for example, can be particularly challenging for groups that have experienced marginalization throughout their lives (like women in this study) to even identify, let alone quantify. As more research stresses the importance of understanding the role of Indigenous-specific social determinants in the health and well-being of Indigenous people in Canada [12], however, this work is important, timely and necessary.

### Strengths and limitations

This study had several strengths. The Cedar Project’s criterion for defining Aboriginal Status was any individual who self-identified as Metis, Aboriginal, First Nations, Inuit, and status and non-status Indians. This type of self-identification, therefore, was more inclusive and was also in keeping with post-colonialism approaches in research. This data set included variables surrounding foster care involvement, residential schooling histories, and sexual abuse questions which allowed for more culturally appropriate and nuanced analyses. The Cedar Project Partnership actively maintains the quality of their data and try to minimize any reporting bias through the extensive training of their Aboriginal interviewers, assurances of confidentiality and availability of support services.

This study also had several limitations. Analysis was limited to previously collected data based on self-report that was cross-sectional, limiting conclusions about causation. Recruitment was non-random and there was no way to determine non-response bias. The limited focus of the study population means that generalizations to the general population of young Aboriginal women could not be made. Finally, it is unclear if women in the study used drugs and/or alcohol during their pregnancies.

## Conclusion

This analysis provided insight into the social contexts of women who have experiences with pregnancy as well as drug and/or alcohol use and highlighted the need to include Indigenous-specific determinants of health when examining young Aboriginal women’s social, political and historical contexts in relation to their experiences with pregnancy and substance use.

## Footnotes

1 Collectively refers to the original people of North America, including the three distinct groups of First Nations (historically referred to as Indian), Metis and Inuit peoples (Constitution Act, 1867). Over 1.4 million individuals in Canada identify themselves as an Aboriginal person (Statistics Canada, 2011).

2 The terms Aboriginal, First Nations, Indigenous, Status Indian and Indian are used in accordance with the term used by the cited authors. Otherwise, the authors use the term Indigenous to acknowledge Indigenous peoples of Canada’s international legal rights under the UN Declaration of the Rights of Indigenous Peoples.

3 The practice of people who are homeless or otherwise socially disadvantaged in society, trading sex for food, a place to sleep, or other basic needs, including drugs.
