# Correction to: The Cedar Project: Using Indigenous-specific determinants of health to predict substance use among young pregnant-involved Indigenous women in Canada

**DOI:** 10.1186/s12905-017-0464-1

**Published:** 2017-11-29

**Authors:** Sana Z. Shahram, Joan L. Bottorff, Nelly D. Oelke, Leanne Dahlgren, Victoria Thomas, Patricia M. Spittal, For the Cedar Project Partnership

**Affiliations:** 1 Faculty of Health and Social Development, University of British Columbia, Kelowna, Canada; 2 Institute for Healthy Living and Chronic Disease Prevention, and School of Nursing, Faculty of Health and Social Development, University of British Columbia, Kelowna, Canada; 3 Faculty of Health Sciences, Australian Catholic University, Melbourne, Australia; 4 School of Nursing, Faculty of Health and Social Development, University of British Columbia, Kelowna, Canada; 5 Department of Obstetrics & Gynaecology, Faculty of Medicine, University of British Columbia, Vancouver, Canada; 6 Wuikinuxv Nation, The Cedar Project, Vancouver, Canada; 7 School of Population and Public Health, University of British Columbia, Vancouver, Canada; 8 Present Address: Postdoctoral Research Fellow, Centre for Addictions Research of British Columbia, University of Victoria, Victoria, Canada

## Correction

After publication of the original article [1] it was noted that the title of this manuscript was incorrect. The title presently reads “The cedar project: using indigenous-specific determinants of health to predict substance use among young pregnant-involved aboriginal women” but should read “The Cedar Project: Using Indigenous-specific determinants of health to predict substance use among young pregnant-involved Indigenous women in Canada”.

The original manuscript was also updated.
